# Holographic molecular binding assays

**DOI:** 10.1038/s41598-020-58833-7

**Published:** 2020-02-06

**Authors:** Yvonne Zagzag, M. Francesca Soddu, Andrew D. Hollingsworth, David G. Grier

**Affiliations:** 1 Department of Physics and Center for Soft Matter Research, New York University, New York, NY 10003 USA; 2 Department of Physics, City College of New York, New York, NY 10031 USA; 3 Present Address: Department of Physics and Astronomy, University of Pennsylvania, Philadelphia, PA 19104 USA

**Keywords:** Assay systems, Optical techniques

## Abstract

We demonstrate that holographic particle characterization can directly detect binding of proteins to functionalized colloidal probe particles by monitoring the associated change in the particles’ size. This label-free molecular binding assay uses in-line holographic video microscopy to measure the diameter and refractive index of individual probe spheres as they flow down a microfluidic channel. Pooling measurements on 10^4^ particles yields the population-average diameter with an uncertainty smaller than 0.5 nm, which is sufficient to detect sub-monolayer coverage by bound proteins. We demonstrate this method by monitoring binding of NeutrAvidin to biotinylated spheres and binding of immunoglobulin G to spheres functionalized with protein A.

## Introduction

**Figure 1:**
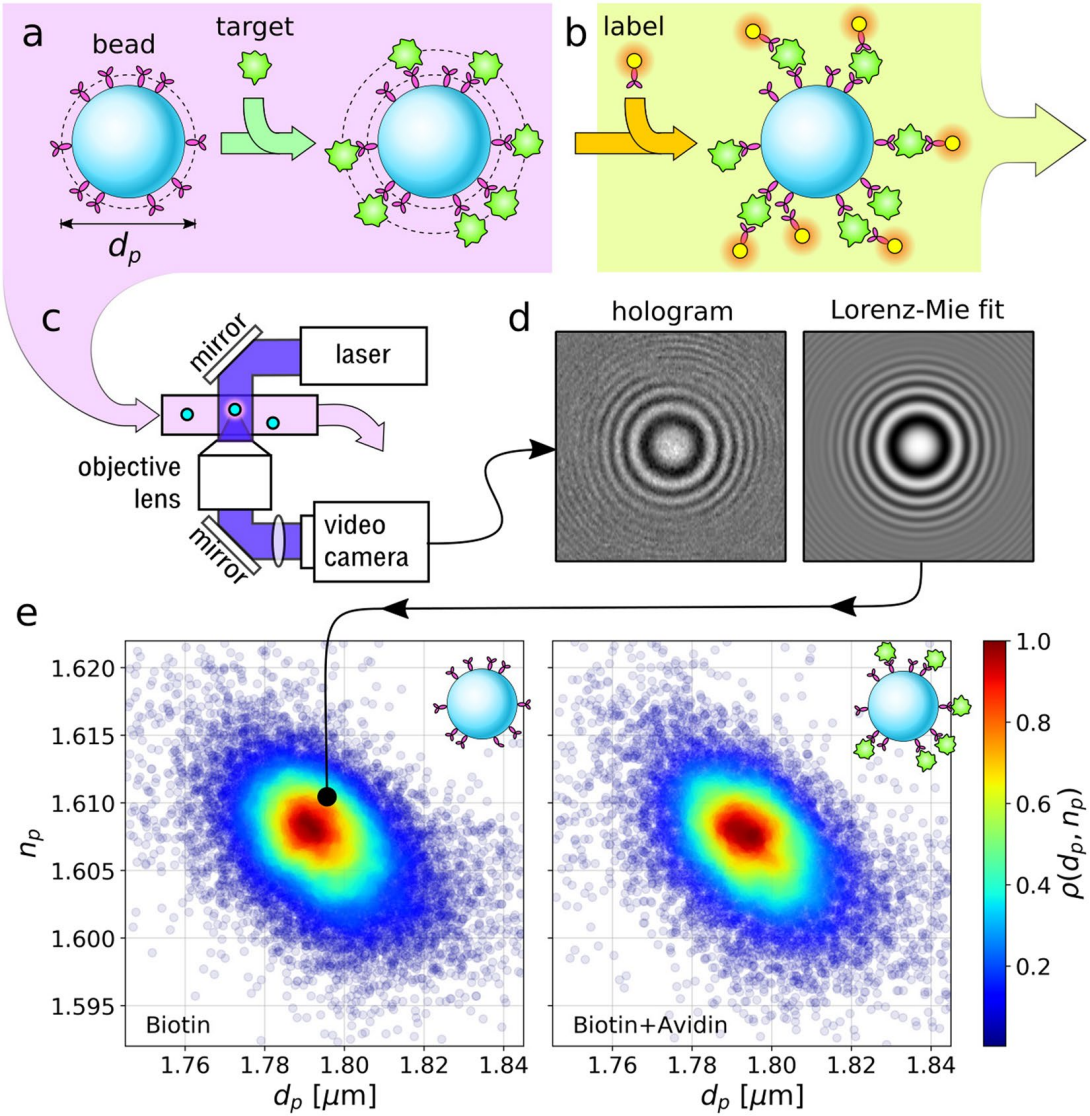
Holographic molecular binding assay. (**a**) Functionalized colloidal spheres are incubated with target molecules that bind to the beads’ surface groups. (**b**) Conventional analysis requires washing and incubation with fluorescent labels that also bind to the surface-bound target molecules. These labels’ fluorescence is read out in a flow cytometer. (**c**) Fluid-borne beads travel down a microfluidic channel where they are illuminated by a collimated laser beam. Scattered light interferes with the rest of the beam to form holograms that are recorded with a video camera. (**d**) Each hologram is fit pixel-by-pixel to predictions of the Lorenz-Mie theory of light scattering to measure the diameter, *d*_*p*_, and refractive index, *n*_*p*_, of the associated sphere. This measurement is sufficiently precise to detect the change in diameter associated with molecular binding. (**e**) Joint probability distribution, *ρ*(*d*_*p*_, *n*_*p*_), of particle diameter and refractive index measurements for biotinylated polystyrene spheres before and after incubation with NeutrAvidin. Each point represents the properties of one bead.

Bead-based molecular binding assays use micrometer-scale colloidal spheres as solid substrates for functional sites to which target molecules can bind. Figure 1(a) illustrates the principle. The beads for these assays can be synthesized in bulk without requiring microfabrication and lend themselves to combinatorial processing. Because of its flexibility and low cost, this approach has been widely adopted for enzyme assays^1^, immunoassays^2^, and assays for gene expression^3^. The challenge in all such applications is to determine whether or not target molecules have bound to functional groups on the beads’ surfaces. The standard approach, illustrated in Fig. 1(b), involves tagging any bound molecules with fluorescent labels and then quantitating the beads’ fluorescence with a flow cytometer. The additional reagents and processing steps required for fluorescence detection increase the cost, complexity, measurement time and skill required for bead-based assays.

Here, we demonstrate that holographic particle characterization^4,5^ can eliminate the extra steps associated with fluorescence detection by directly measuring the increase in the beads’ diameters caused by target molecules binding to their surfaces. The measurement technique is illustrated schematically in Fig. 1(c). High-speed automated fitting of in-line holographic microscopy images such as the example in Fig. 1(d) yields precise measurements of the probe beads’ diameters before and after they are incubated with the target molecule. Interpreting holographic characterization data with the effective-sphere model^6–8^ and rigorous statistical methods^9^ yields a label-free assay that is fast, cost-effective and sensitive. We demonstrate this method through measurements on two model systems: avidin binding to biotinylated polystyrene spheres, and immunoglobulin G (IgG) binding to polystyrene spheres functionalized with protein A.

Figure 1(e)shows typical holographic characterization results for a sample of 20106 biotinylated polystyrene spheres before incubation with NeutrAvidin, and 14612 after. Each plot symbol represents the diameter, *d*_*p*_, and refractive index, *n*_*p*_, of a single bead. The points are colored by the relative density of measurements, *ρ*(*d*_*p*_, *n*_*p*_), computed with a Gaussian kernel density estimator^10^.

**Figure 2:**
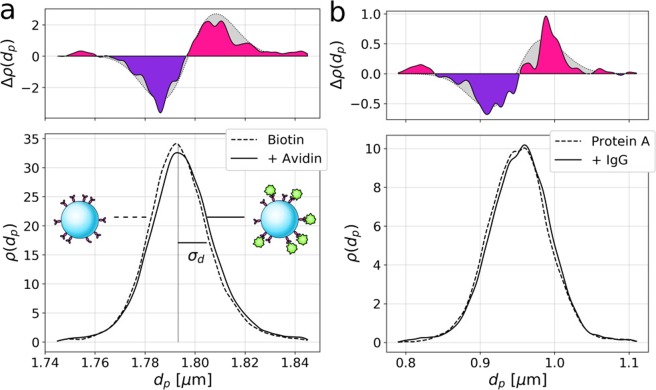
(**a**) Projected distribution *ρ*(*d*_*p*_) of particle diameters for biotinylated polystyrene spheres before and after incubation with NeutrAvidin. The width of the distribution, *σ*_*d*_, reflects the population polydispersity in diameter. The difference, *Δρ*(*d*_*p*_), between these distributions is consistent with a statistically significant increase of *Δd*_*p*_ = (1.4 ± 0.1) nm in the mean particle diameter. (**b**) Analogous data for IgG binding to spheres coated with protein A shows an increase of *Δd*_*p*_ = (3.2 ± 0.4) nm.

Although the probe particles are nominally monodisperse, their size distribution, $\rho ({d}_{p})={\int }_{0}^{\infty }\rho ({d}_{p},{n}_{p})\ d{n}_{p}$, plotted in Fig. 2(a), has a half-width at half maximum of *σ*_*d*_ = 11 nm, which is greater than the 8 nm maximum extent of NeutrAvidin^11^. The small contribution of bound NeutrAvidin to the size of the beads can be resolved, nevertheless, by monitoring the shift in the mean diameter in a sufficiently large population of spheres.

The population-average diameter of the biotinylated probe spheres is *d*_*p*_ = (1.7933 ± 0.0001) *μ*m, including the statistical uncertainty in the mean of 0.1 nm. After incubation with NeutrAvidin, the holographically measured mean diameter increases to (1.7947 ± 0.0001) *μ*m, a shift of *Δd*_*p*_ = (1.4 ± 0.1) nm. This increase is statistically significant. Welch’s t-test rejects the null hypothesis that the distributions have equal means with a p-statistic of 9.3 × 10^−14^. The Kruskal-Wallis test similarly yields a p-statistic of 1.1 × 10^−16^ and the Kolmogorov-Smirnov two-sample statistic for the two distributions is 2.3 × 10^−14^.

The shift in the particles’ average diameter post-binding is clearly resolved in the difference of the two diameter distributions,

1 $$\Delta \rho ({d}_{p})={\rho }_{{\rm{avidin}}}({d}_{p})-{\rho }_{{\rm{biotin}}}({d}_{p}),$$

that also is plotted in Fig. 2(a). The gray-shaded region shows the expected signal for two normal distributions displaced by the measured difference in mean diameter.

 Figure 2(b) shows the corresponding assay for immunoglobulin G (IgG) binding to probe spheres coated with protein A. These distributions were compiled from measurements on 18930 spheres before incubation and 14667 after. In this case, the observed increase in the population-averaged diameter is *Δd*_*p*_ = (3.2 ± 0.4) nm, which is substantially larger than the 1 nm increase obtained with NeutrAvidin. This shift also is statistically significant, with Welch’s t-test yielding a p-statistic of 1.8 × 10^−6^, Kruskal-Wallis yielding a p-statistic of 1.9 × 10^−9^ and Kolmogorov-Smirnov yielding a p-statistic of 5.7 × 10^−8^.

## Materials and Methods

### Biotinylated polystyrene spheres

Monodisperse polystyrene spheres are functionalized with a covalently linked monolayer of biotin to create biotinylated probe particles. Stock polystyrene colloidal spheres are obtained from a commercial source (Magsphere, Inc., product number AM002UM, lot number AM3791-0618). The manufacturer specifies that these beads have a nominal diameter of *d*_*p*_ = (1.83 ± 0.09) *μ*m. Holographic characterization yields a mean diameter of *d*_*p*_ = (1.7933 ± 0.0001) *μ*m, which is consistent with this specification. The distribution of diameters, however, has a half width at half maximum of *σ*_*d*_ = 11 nm, which is substantially smaller than suggested by the specification. We conclude that the actual polydispersity in diameter of this sample is somewhat less than 2%, which is beneficial for the holographic molecular binding assay because the narrow size distribution facilitates precise measurement of small changes in the population-average diameter.

Each sphere has approximately 10^7^ reactive amidine groups on its surface, each of which is a potential attachment site for biotin. As delivered, these sites are blocked by ionic surfactants and other stabilizers. Spheres are prepared for functionalization by dialysis against 1 M acetic acid (pH = 2.4) to remove these additives and are redispersed in a PBS buffer solution adjusted to pH 7.4, with an ionic strength of 63 mM. Because the charge on the spheres’ primary amine groups varies with pH, we stabilize the spheres against aggregation by adding 0.1 wt% F-108 (Aldrich, product number 542342-1 KG, lot number MKBP4463V), an adsorbing non-ionic triblock copolymer surfactant. Electrophoresis with a Malvern Zetasizer Nano ZS confirms that the stabilized particles are positively charged under acidic conditions and negatively charged at neutral pH due to protonation and deprotonation of the amine groups, respectively.

Biotin is attached to the spheres’ primary amine groups through reaction with an N-hydroxy sulfosuccinimide ester of biotin, specifically the doubly-long-chained succinimidyl-6-(biotinamido)-6-hexanamido hexanoate (NHS-LC-LC-Biotin, Thermo Scientific; product number 21343, lot number TE262577). We use the NHS-LC-LC-Biotin as delivered and prepare it by dissolving in N,N-dimethylformamide (DMF) (Sigma Aldrich; product number 227056 Lot No. SHBJ2091) at 50 mg mL^−1^. With mixing, 1 mL of the biotin solution is combined with 70 mL of the colloidal dispersion at room temperature and the subsequent reaction is allowed to proceed for 1 h. The unreacted portion of the biotin compound is subsequently washed out. The biotinylated spheres finally are redispersed in PBS buffer solution at a concentration of 10^9^ particles/mL.

The biotin coverage of the functionalized spheres is assayed by titrimetric spectrophotometry at 500 nm using 2-(4-hydroxyphenylazo)benzoic acid (HABA, Thermo Scientific; product number 28010, lot number BCBV7265), a fluorescent dye that binds to avidin and is displaced when avidin binds to biotin^12^. These measurements were validated by control measurements of HABA displacement by hexahydro-2-oxotheno(3,4-d)-imidazole-4-pentanoic acid (D(+)-biotin, Acros Organics, product number 230095000, lot number A0381899). The results are consistent with an areal density of 1 accessible biotin molecule per 6 nm^2^ on the spheres’ surfaces.

### NeutrAvidin binding to biotinylated polystyrene spheres

NeutrAvidin is a 60 kDa biotin-binding protein that has been deglycosylated to suppress non-specific binding^13^. Avidin shows a high affinity for biotin, with an association constant on the order of 10^15^ M^−1^. Each avidin tetramer can bind up to four biotin molecules.

NeutrAvidin is obtained as a powder from a commercial source (Thermo Scientific, product number 31000, lot number UA276095) and is reconstituted into PBS buffer adjusted to pH 7.4 at a total ionic strength of 63 mM with 0.1 wt% F-108 surfactant. The final concentration of protein is 54 *μ*g mL^−1^.

Biotinylated polystyrene spheres were incubated with NeutrAvidin by adding one part per thousand of the colloidal dispersion to the NeutrAvidin solution. This yielded a concentration of 10^6^ particles/mL.

### Protein A coated polystyrene spheres

Protein A is a 42 kDa protein that plays multiple roles in bacterial pathogenicity. The immune response to protein A includes strong binding by immunoglobulin G (IgG), one of a family of naturally occurring antibodies. Polystyrene beads coated with protein A by passive adsorption were obtained commercially (Bangs Laboratories, Inc.; catalog number CP02000, lot number 13597). These spheres are reported by the manufacturer to have a mean diameter of *d*_*p*_ = 0.989 *μ*m with the distribution of diameters falling in the range 0.95 *μ*m ≤ *d*_*p*_ ≤ 1.05 *μ*m. Holographic characterization yields a mean diameter of *d*_*p*_ = (0.9518 ± 0.0003) *μ*m in a distribution with half width at half maximum of *σ*_*d*_ = 37 nm. Each milligram of spheres is specified to have a binding capacity of 13.77 *μ*g of IgG.

The affinity of protein A for IgG depends on pH, with strong biding occurring at high pH (8.2) and weak, reversible binding occurring at low pH (2.8). Probe spheres coated with protein A are stored at 1% volume fraction in a 50 mM sodium borate antibody binding buffer (ABB) with a pH of 8.2. The dispersion is diluted for use to a concentration of 10^6^ particles/mL with ABB.

### Incubation with IgG

Rabbit IgG in solution at a concentration of 10 mg mL^−1^ was obtained commercially (EMD Millipore Corp.; catalog number PP64, lot number 3053798). The stock solution is diluted for use to a concentration of 20 *μ*g mL^−1^ in ABB, which corresponds to a 10× excess given the spheres’ binding capacity. One sample of spheres serves as the reference and is diluted with ABB alone. The other sample is diluted with ABB containing IgG. Both samples are gently agitated at room temperature before holographic characterization measurements are performed.

### Holographic particle characterization

Holographic particle characterization uses a variant of in-line holographic video microscopy that illuminates the sample with a collimated laser beam^4,14^, as illustrated in Fig. 1(c). Light scattered by a colloidal particle interferes with the rest of the beam in the microscope’s focal plane. The microscope magnifies the interference pattern and relays it to a video camera that records its intensity. Each video frame constitutes a hologram of the particles in the observation volume. A typical region of interest capturing the hologram of a single polystyrene sphere is also is presented in Fig. 1(d).

Treating the illumination as a monochromatic linearly polarized plane wave with electric field

2 $${\bf{E}}({\bf{r}})={E}_{0}{e}^{ikz}\hat{x},$$

the measured hologram for a particle at position **r**_*p*_ may be modeled as^4^

3 $$I({\bf{r}})={E}_{0}^{2}| \hat{x}+{e}^{ik{z}_{p}}{{\bf{f}}}_{s}(k({\bf{r}}-{{\bf{r}}}_{p})){| }^{2},$$

where *k* is the wavenumber of the light in the medium and **f**_*s*_(*k***r**) is the Lorenz-Mie scattering function^15,16^, which is parameterized by the particle’s diameter, *d*_*p*_, and refractive index, *n*_*p*_. Fitting a measured hologram pixel-by-pixel to Eq. 3 yields measurements of *d*_*p*_ and *n*_*p*_ for the associated particle in addition to its three-dimensional position, **r**_*p*_^4^. The result of such a fit is shown in Fig. 1(d).

Holographic particle characterization has been demonstrated for colloidal particles in the size range 0.5 *μ*m ≤ *d*_*p*_ ≤ 10 *μ*m at concentrations in the range 10^3^ particles/mL to 10^7^. Validation measurements demonstrate that the precision for an individual particle size measurement is *Δd*_*p*_ = 5 nm and the precision for the refractive index is *Δn*_*p*_ = 0.003^17^. The same fits also yield the in-plane position to within 1 nm across the microscope’s field of view and the axial position to within 3 nm over a range extending to 100 *μ*m^17^. Populations of particles can be analyzed by streaming a fluid sample through the microscope’s observation volume in a microfluidic channel, as illustrated in Fig. 1(c). Each particle can be detected and analyzed multiple times as it passes through the observation volume to further reduce the uncertainties in its properties^5^.

**Figure 3:**
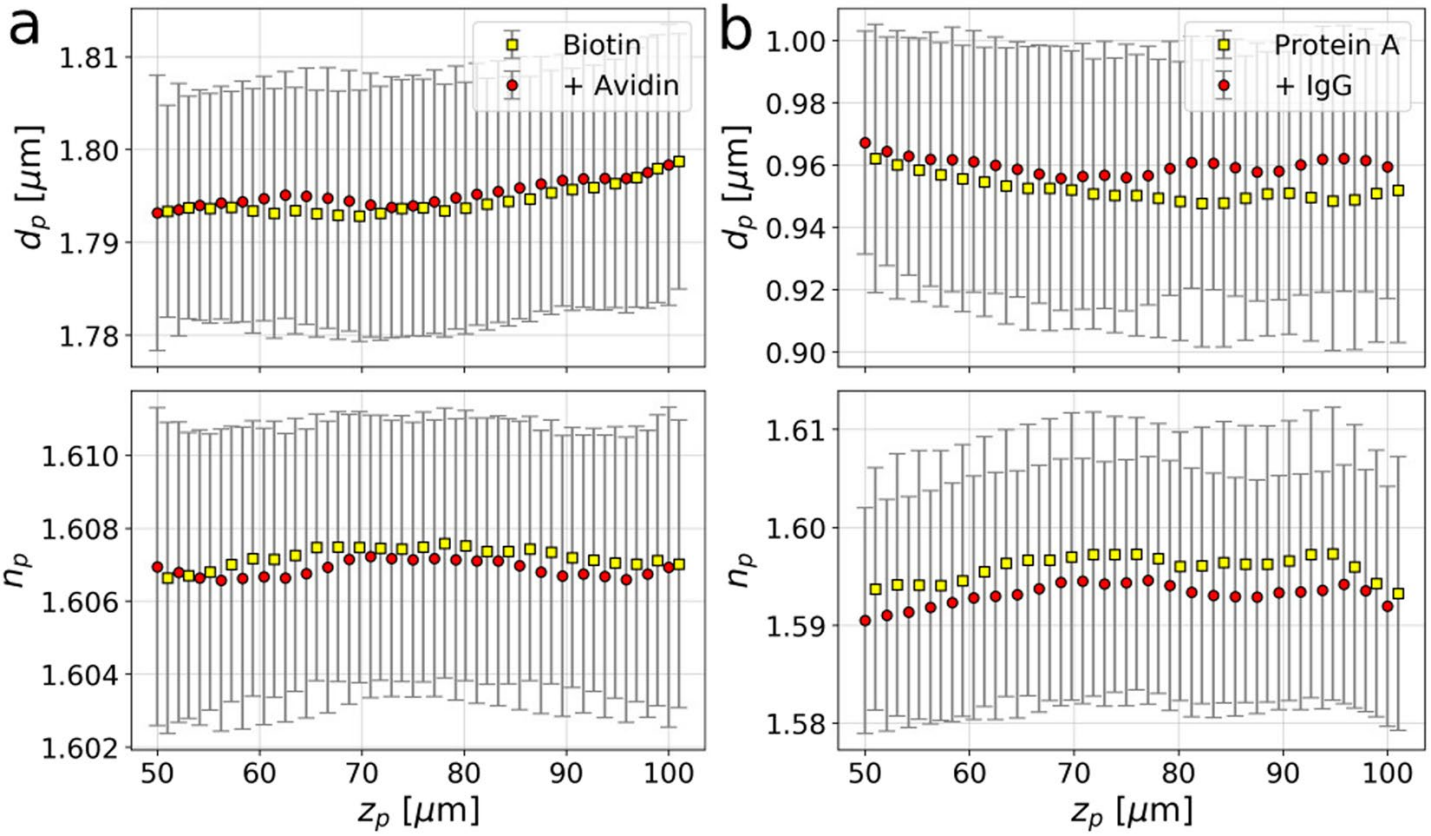
Dependence of holographically measured diameter, *d*_*p*_, and refractive index, *n*_*p*_, on particle position, *z*_*p*_, within the sample cell for (**a**) biotinylated spheres before (yellow squares) and after (red circles) binding by NeutrAvidin and (**b**) spheres coated with Protein A before and after binding by IgG. Population averages and standard deviations are calculated at each height using kernel density estimators.

Different particles pass through the imaging volume at different heights, *z*_*p*_, relative to the microscope’s focal plane. Although Eq. (3) accounts for the axial position, subtle height-dependent imaging mechanisms can influence results for *d*_*p*_ and *n*_*p*_^18^. Figure 3 shows how the characterization data plotted in Figs. 1 and 2 vary with *z*_*p*_. Population averages (discrete points) and standard deviations (error bars) are estimated at each height with kernel density estimators^10^. The range of axial positions represents the 50 *μ*m depth of the xCell microfluidic channels used for these measurements.

The populations’ mean diameters are systematically larger after incubation with target molecules. This is consistent with the statistically significant shifts reported in Fig. 2. At the same time, the measured refractive indexes are slightly but systematically smaller after binding. The biotinylated spheres, Fig. 3(a), have a mean refractive index, *n*_*p*_ = (1.607 ± 0.003) that is consistent with expectations for polystyrene at the imaging wavelength, *λ* = 447 nm^19^. The smaller polystyrene spheres used as a substrate for Protein A, Fig. 3(b), have a lower mean refractive, *n*_*p*_ = (1.596 ± 0.011). In this case, the mean refractive index decreases systematically by 0.003 after incubation with IgG. This small shift suggests that the thicker protein coating affects the sphere’s optical properties beyond simply increasing their size.

Equation (3) can be generalized to accommodate coated spheres and core-shell particles^20^. This approach has been used successfully to characterize colloidal microshells whose core and shell both have dimensions comparable to the wavelength of light and whose refractive indexes differ substantially from each other^21^. In the present case, however, the molecular-scale coating is much thinner than the wavelength of light, and its refractive index differs only slightly from the refractive index of the core particle. We expect, therefore, that corrections to the effective-sphere model’s predictions due to the coated particles’ core-shell structure cannot be resolved with our instrument, although the associated changes in size can be resolved.

Unlike other cytometric techniques for high-resolution particle sizing^22^, holographic particle characterization does not require calibration with size standards. The only instrumental parameters are the laser wavelength, the microscope’s magnification and the refractive index of the fluid medium. Similarly, fitting to the generative model from Eq. (3) rather than computing phenomenological metrics^23^ eliminates the need for per-particle calibrations.

### Measurement with xSight

Holographic particle characterization measurements are carried out with a Spheryx xSight, a commercial instrument that automatically analyzes populations of colloidal particles. A 30 *μ*L aliquot of the sample to be measured is introduced into one of the eight sample reservoirs of a disposable xCell microfluidic chip that is mounted on the xSight’s sample stage. Up to 3 *μ*L of the sample is transported through the observation volume by a pressure-driven flow for analysis. The entire measurement is completed in 20 min and reports the properties of roughly 5000 particles assuming typical concentrations of 10^6^particles/mL. The data sets presented in Figs. 1(e) and 2 are each accumulated from three such measurements. The six measurements required for an assay therefore can be completed in two hours.

### Effective-sphere interpretation

Binding molecules to the surface of a sphere increases the sphere’s apparent diameter from its bare value of *d*_0_ to its coated value of *d*_*p*_, as measured by holographic microscopy. The actual coverage of molecules generally does not take the form of a continuous film, but rather resembles bumps on the surface of the original sphere. In the effective-sphere model^6,7^, these asperities are treated as a continuum whose refractive index is intermediate between that of the molecules and that of the medium. Complete occupancy of the binding sites increases the sphere’s effective radius by an amount, *δ*, that is likely to be smaller than the diameter of a target molecule because of gaps between binding sites. If target molecules occupy a fraction, *f*, of binding sites, the diameter reported by holographic microscopy should increase to

4 $${d}_{p}={d}_{0}+2\delta \ \sqrt{f}.$$

The observed increase in mean diameter therefore should be smaller than twice the size of the target molecule.

### Statistical analysis

Each molecular binding assay is performed with three independent measurements of the functionalized beads after incubation in buffer and another three separate measurements of the probe beads incubated with target molecules. Each measurement is performed with a separate 30 *μ*L aliquot of sample in one channel of an eight-channel xCell sample chip. The chip is removed from the instrument for filling and then immediately reseated for each measurement. We use the measured shape of the flow profile^5^ to ensure that the sample cell is seated reproducibly in the optical train for each run. Repeating characterization measurements allows us to assess reproducibility of the results from run to run, thereby ensuring that an observed shift in the mean diameter reflects a change in the sample rather than instrumental variations. The original report on holographic molecular binding assays^5^ did not control for instrumental variability.

The three data sets obtained from samples without target molecules are tested to establish that all are derived from the same underlying distribution of particle sizes. Specifically, they are compared with Welch’s t-test, the Kruskal-Wallis H-test for independent samples and the Kolmogorov-Smirnov two-sample test. All statistical tests are performed in the Python programming language with routines from the scipy.stats library. The same battery of tests is performed on data acquired from the three samples incubated with target molecules.

Having confirmed that the two groups of measurements, with and without target molecules, are internally self-consistent, we compile each group into a combined data set. We then use the same statistical tests to compare the two combined data sets. This time, the goal is to ascertain whether or not the two data sets are drawn from different size distributions. The data presented in Figs. 1 and 2 satisfy all of these tests.

## Results and Discussion

We have demonstrated that holographic particle characterization can monitor molecular binding to functionalized beads by directly detecting the associated change in the beads’ size, without additional labeling or processing. The measurement relies on the ability of holographic microscopy to resolve the diameter of an individual colloidal sphere with nanometer-scale precision. Combining thousands of single-bead measurements then resolves the population-average bead diameter with sub-nanometer precision, which is sufficient to resolve even sub-monolayer coatings of macromolecular targets.

Statistical analysis of such population-based studies can distinguish small but real shifts in the probe spheres’ mean diameters from statistical fluctuations due to the beads’ broad size distribution. This critical step was not performed in a previous proof-of-concept demonstration^5^.

The need for statistical analysis of holographic molecular binding measurements could be eliminated by monitoring changes in the size of a single probe bead before and after the introduction of an analyte. Single-bead label-free measurements would lend themselves to multiplexed assays because different types of probe beads can be distinguished both by size and by refractive index^24^. Such measurements will be described elsewhere.

## Data Availability

All materials, data and software discussed in this publication are available by request from the corresponding author.
